# E_μ_ and 3′RR IgH enhancers show hierarchic unilateral dependence in mature B-cells

**DOI:** 10.1038/s41598-017-00575-0

**Published:** 2017-03-27

**Authors:** A. Saintamand, C. Vincent-Fabert, M. Marquet, N. Ghazzaui, V. Magnone, E. Pinaud, M. Cogné, Y. Denizot

**Affiliations:** 1 CNRS UMR 7276, CRIBL, Université de Limoges, Limoges, France; 2 CNRS et Université de Nice Sophia Antipolis, Institut de Pharmacologie Moléculaire et Cellulaire, UMR 6097, Sophia, Antipolis France; 3 INSERM U1236, Université Rennes 1, Rennes, France

## Abstract

Enhancer and super-enhancers are master regulators of cell fate. While they act at long-distances on adjacent genes, it is unclear whether they also act on one another. The immunoglobulin heavy chain (IgH) locus is unique in carrying two super-enhancers at both ends of the constant gene cluster: the 5′E_μ_ super-enhancer promotes VDJ recombination during the earliest steps of B-cell ontogeny while the 3′ regulatory region (3′RR) is essential for late differentiation. Since they carry functional synergies in mature B-cells and physically interact during IgH locus DNA looping, we investigated if they were independent engines of locus remodelling or if their function was more intimately intermingled, their optimal activation then requiring physical contact with each other. Analysis of chromatin marks, enhancer RNA transcription and accessibility in E_μ_- and 3′RR-deficient mice show, in mature activated B-cells, an unilateral dependence of this pair of enhancers: while the 3′RR acts in autonomy, E_μ_ in contrast likely falls under control of the 3′RR.

## Introduction

**Figure 1:**
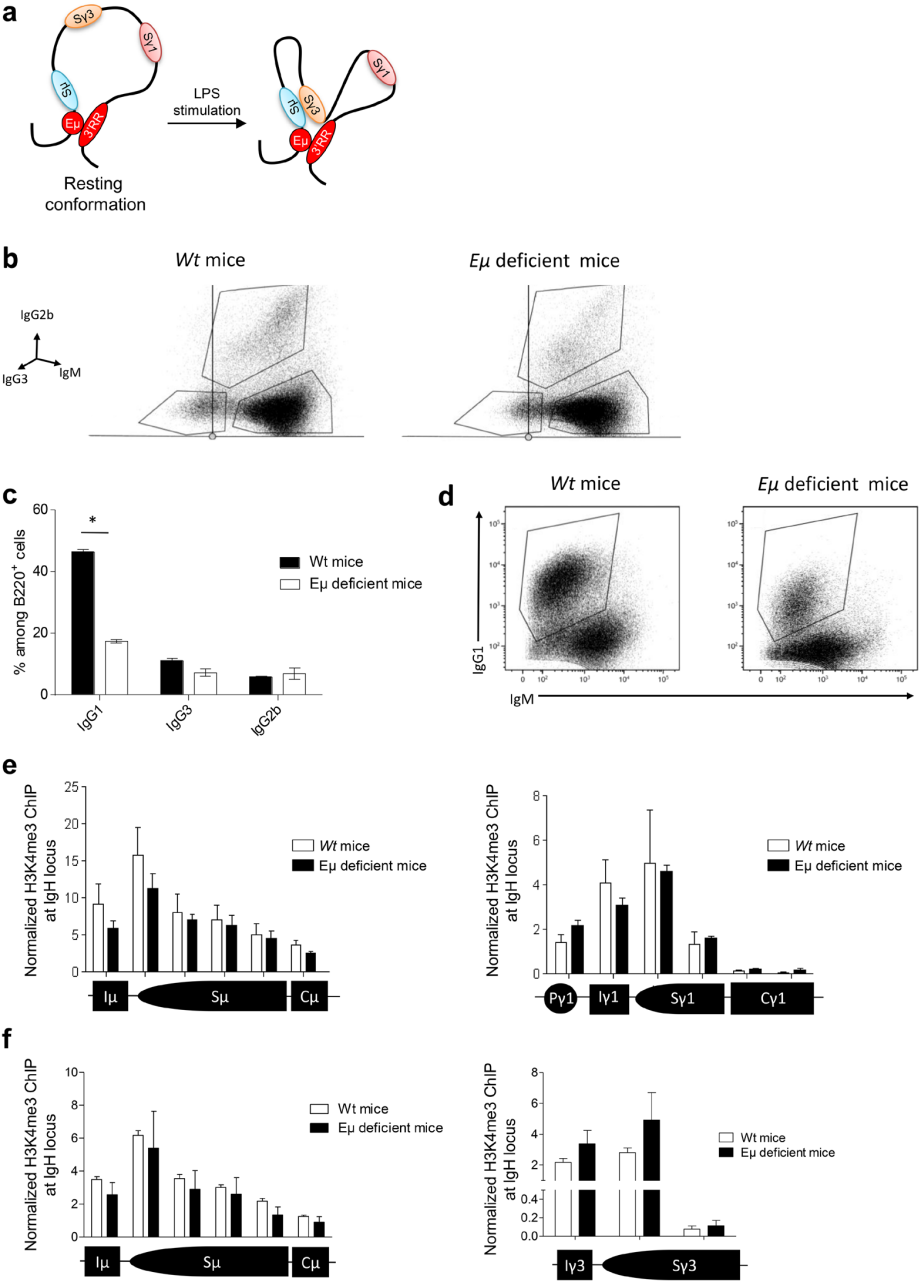
E_μ_ and CSR. (**a**) Schematic 3D conformation of the IgH locus during LPS-induced CSR. 3C experiments indicated that E_μ_ and the 3′RR are in close proximity in resting B cells. After appropriated stimulation the activated S acceptor region gets closer the S_μ_ donor region. Adapted from Wuerffel *et al*.^4^. (**b**) Flow cytometry analysis of IgG_3_ and IgG_2b_ CSR in E_µ_-deficient B-cells. Cells were stimulated 4 days with LPS. Cells were labelled with anti-B220-BV421, anti-IgG_3_-FITC, anti-IgG_2b_-PE and anti-IgM-PC7 antibodies. Cells were gated on B220^+^ B-cells. One representative experiment out of four is shown. (**c**) Quantitative analysis of IgG_1_, IgG_3_ and IgG_2b_ CSR. Results are reported as mean ± SEM of 4 independent experiments. *p < 0.05, Mann-Whitney *U*-test. (**d**) Flow cytometry analysis of IgG_1_ CSR in E_μ_-deficient B-cells. Cells were stimulated 4 days with LPS + IL-4. Cells were labelled with anti-B220-BV421, anti-IgG_1_-PE and anti-IgM-PC7 antibodies. Cells were gated on B220^+^ B-cells. One representative experiment out of four is shown. (**e**) H3K4me3 epigenetic mark in *S*_μ_ and *S*_γ1_ during IgG_1_ CSR ChIP assays were performed with CD43^−^ splenic B-cells from E_μ_-deficient and *wt* mice. Cells were stimulated with LPS + IL-4 for 2 days. Background signals from mock samples with irrelevant antibody were subtracted. Values were normalized to the total input DNA. Data are the mean ± SEM of 3 independent experiments with 2 mice. (**f**) H3K4me3 epigenetic mark in *S*_μ_ and *S*_γ3_ during IgG_3_ CSR. Cells were stimulated with LPS for 2 days. Same protocol as in part E.

Super-enhancers (SEs) are master regulators of cell fate which differ from basic enhancers by their ten-fold higher load of chromatin marks, their binding of mediator, their long length and their impact on nuclear organisation^1, 2^. The immunoglobulin heavy chain (IgH) locus undergoes multiple changes along B-cell differentiation, affecting transcription and accessibility for V(D)J recombination, somatic hypermutation (SHM) and class switch recombination (CSR)^3^. The IgH locus is somehow unique in carrying two SEs, E_μ_ and the 3′ regulatory region (3′RR) at both ends of the constant gene cluster, which control locus remodelling along B-cell differentiation^3^. In mature B-cells, the IgH locus assumes an enigmatic loop conformation in which these two SEs are brought in close proximity despite their 200 kb distance on the chromosome (Fig. 1a)^4^. Two mechanistic hypothesises may explain the function of this 3D chromatin structure: a bidirectional crosstalk between these two SEs allowing reciprocal activation, or a simple chromatin arch assembly that brings enhancers, promoters and switch (S) regions into close proximity to facilitate transcription, accumulation of RNA pol II, AID targeting and S junction machinery. In a third hypothesis, the E_μ_-3′RR interaction might lack any mechanistic role by passively witness transitional links, with E_μ_ on one side promoting S_μ_-S_x_ synapsis and the 3′RR stimulating the I_x_-S_x_ transcriptional unit. We investigated if these two IgH SEs were independent engines of locus remodelling simply combining their proper actions or if their functions were more strongly intermingled and required physical contact with each other. SEs are potent clusters of transcriptional enhancers and regulate the expression of key cell lineage specific genes. The 5′E_μ_ SE has clearly such a role in early developmental stages of B-cells through its key role on V(D)J recombination. Since 5′E_μ_ may be considered as a SE only in pro-B/pre-B-cells but not in mature ones, we used the qualifier of enhancer to name 5′E_μ_ at the mature B-cell stage during this study.

## Results

### E_μ_ and CSR

During CSR, a chromatin loop is found with E_μ_ and 3′RR in close proximity^3, 4^. It is generally accepted that E_µ_ is dispensable for CSR, although some studies suggest that its deletion impacts at minor levels CSR efficiency^5–7^. In this study, flow cytometry analysis shows normal proportion of E_μ_-deficient γ3- and γ2b-expressing B-cells in response to LPS stimulation (Fig. 1b and c). In contrast, the significant decrease of E_μ_-deficient γ1-expressing B-cells in response to LPS + IL4 (Fig. 1c and d) could mostly be attributed to the lack of follicular B-cells previously reported in this model^7^. These results are in accordance with the lowered serum IgG_1_ levels but normal IgG_3_/IgG_2b_ levels in this model^7^. Specific epigenetic mark enrichment in S regions is a prerequisite for CSR^8, 9^. During CSR, the 3′RR SE fosters H3K4me3 histone modifications in the S acceptor but not S_μ_ donor region^10^, suggesting another *cis*-transcriptional enhancer for this role. This role is not devolved to E_μ_ since its deletion did not affect H3K4me3 histone modifications in S_μ_ and S_γ1_ in response to LPS + IL-4 stimulation (Fig. 1e) nor in S_μ_ and S_γ3_ in response to LPS stimulation (Fig. 1f). Altogether, these data indicate that the E_μ_ enhancer is mostly dispensable for CSR and did not poise S_μ_ for CSR. The *cis*-transcriptional enhancer that poised S_μ_ for CSR is currently unknown. One might suggest the enigmatic transcriptional enhancer located between C_γ1_ and C_γ2b_ found to interact with both E_μ_ and 3′RR in pro-B cells^2, 11, 12^.

### E_μ_, 3′RR and respective epigenetic marks

**Figure 2:**
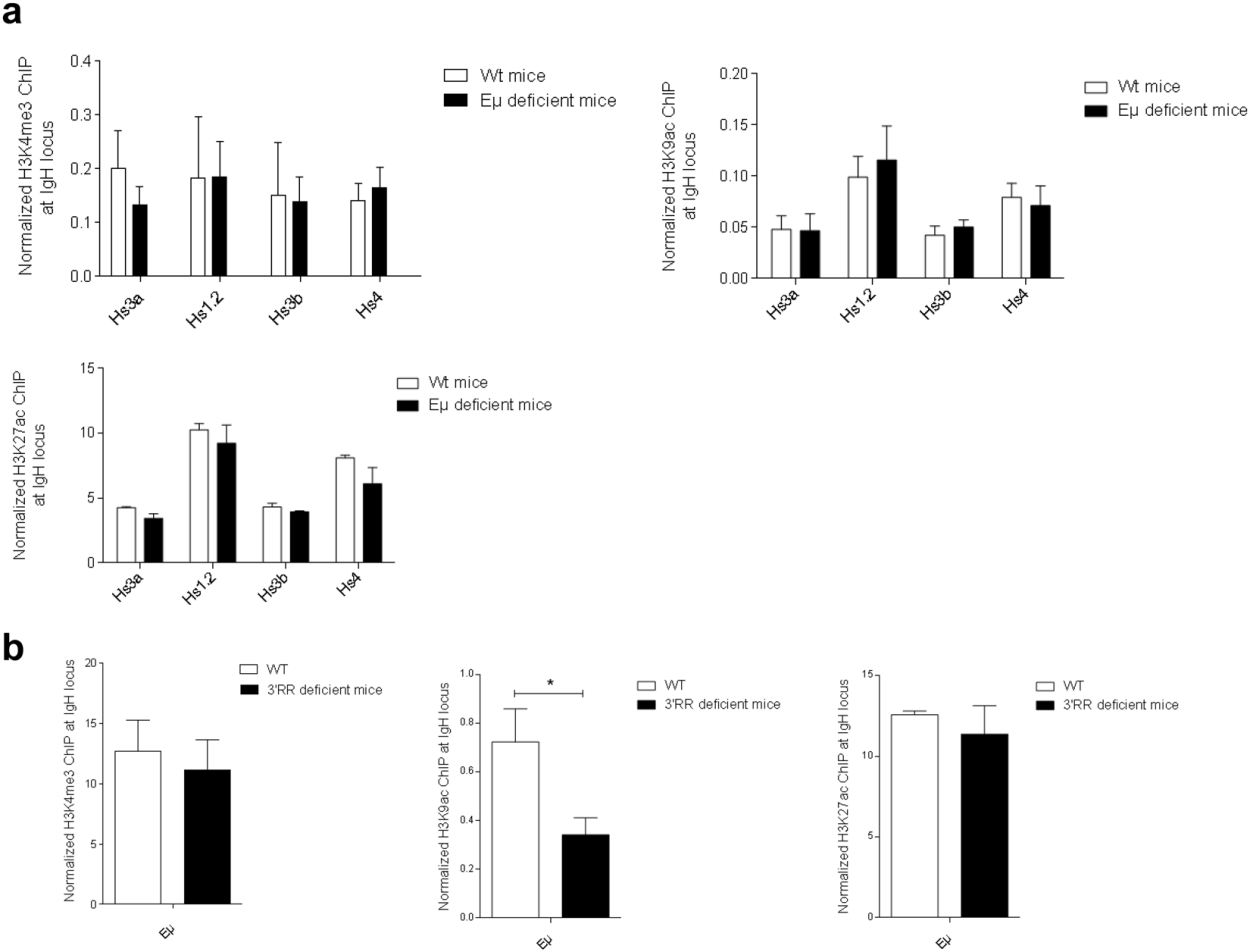
Epigenetic marks in E_μ_ and 3′RR during CSR. (**a**) H3K4me3, H3K9ac and H3K27ac epigenetic marks in the four enhancers (hs3a, hs1,2, hs3b and hs4) of the 3′RR in LPS-stimulated B-cells of E_μ_-deficient and *wt* mice. Cells were stimulated with 5 μg/ml LPS for 2 days. Data are the means ± SEM of 3 independent experiments with 2 mice. (**b**) H3K4me3, H3K9ac and H3K27ac epigenetic marks in E_μ_ in LPS-stimulated B-cells of 3′RR-deficient and *wt* mice. Same experimental protocol as in part (**a**). Data are the means ± SEM of 3 independent experiments with 2 mice. *p < 0.05 (Mann-Whitney *U*-test).

Epigenetic changes in E_μ_ and 3′RR are of importance during CSR^13^. In this study we have focussed on H3K4me3, H3K9ac and H3K27ac epigenetic marks that are associated with active regulatory regions^14^. Lack of the E_μ_ enhancer did not affect H3K4me3, H3K9ac and H3K27ac epigenetic marks in the four 3′RR transcriptional enhancers (hs3a; hs1,2; hs3b; hs4) during LPS-induced B-cell CSR (Fig. 2a). In turn, if deletion of the 3′RR SE had no effect on H3K4me3 and H3K27ac marks of E_μ_ (Fig. 2b), a significant decrease was found for H3K9ac. This difference between epigenetic marks may be explained by the proposed model of sequential histone modifications. Indeed, several studies suggest that tri-methylation of H3K4 is the earliest modification, and that H3K4me3 then facilitate H3 acetylation and thus establishment of chromatin openness. All these marks together may then positively regulate transcription and enhancer activation^15, 16^. At the mature B-cell stage, data suggest the lack of E_μ_-dependence for the 3′RR SE during CSR, and the influence of the 3′RR on the E_μ_ enhancer, a result that fits well with their different kinetics of activation, *i.e*., at the immature and mature B-cell stages for Eμ and 3′RR, respectively^3^.

### Mutual activation of transcription of E_μ_ and 3′RR SEs

**Figure 3:**
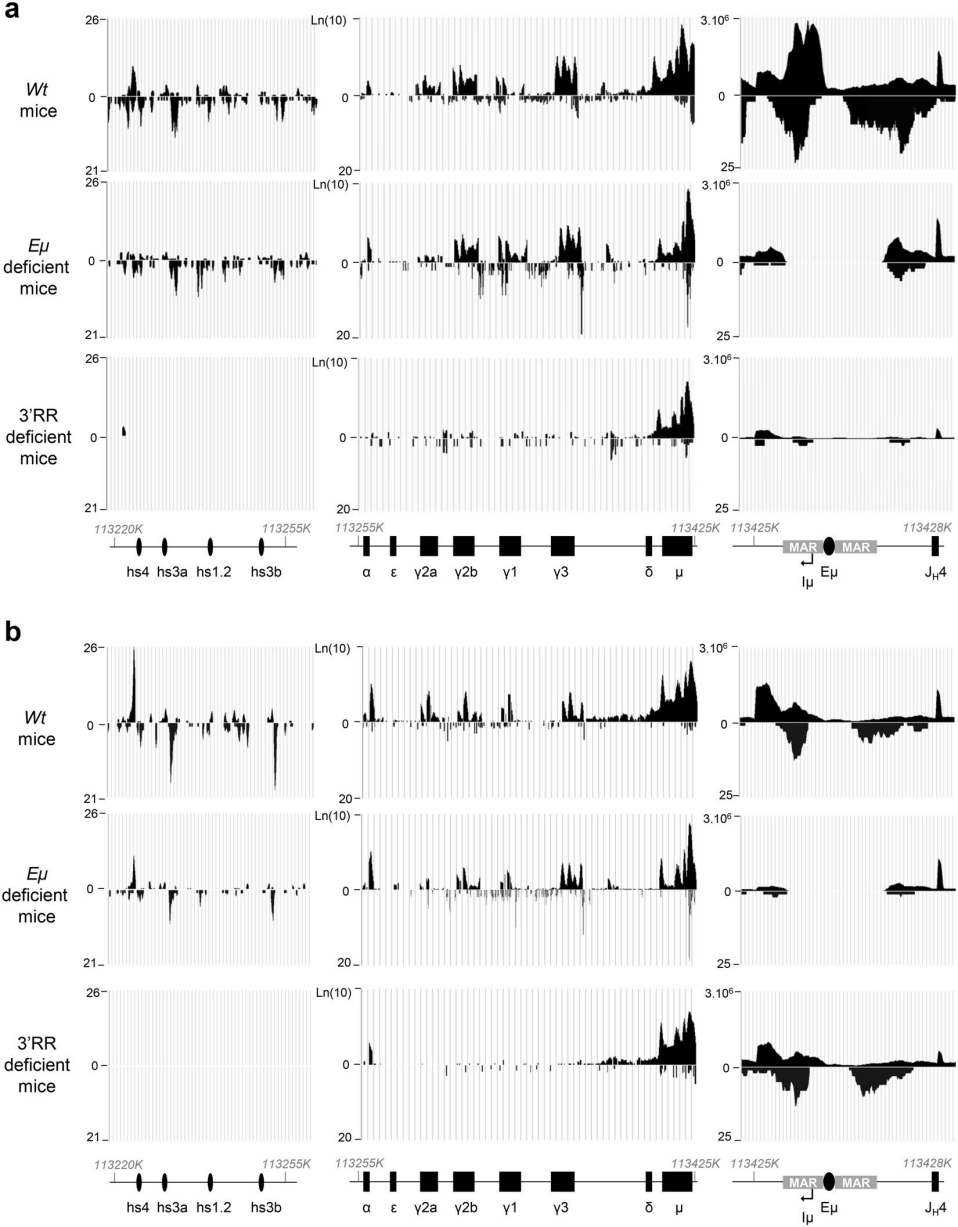
Influence of E_µ_ and 3′RR on IgH transcription during CSR. (**a**) IgH transcription on LPS-stimulated B-cells of *wt*, Eμ-deficient and 3′RR-deficient mice. CD43-depleted splenocytes were cultured for 2 days with 5 μg LPS. RNAseq experiments were done after depletion of rRNA. Data are the mean of two independent experiments with 3 mice per genotype. (**b**) IgH transcription on resting B-cells of *wt*, Eμ-deficient and 3′RR-deficient mice. RNAseq experiments were done with CD43-depleted splenocytes after depletion of rRNA. Data are the mean of two independent experiments with 3 mice per genotype. Precise locations of I_μ_, S_μ_, C_μ_, C_δ_, I_γ3_, S_γ3_, Iγ_2b_ and S_γ2b_ are reported in Supplementary Figs 1–3.

The 3′RR SE controls CSR by acting on germline transcription and histone modifications^8, 17^, that are hallmarks of CSR accessibility. We investigated the effect of the lack of the E_μ_ enhancer or of the 3′RR SE on IgH constant gene transcription units (C_H_) in response to LPS-induced stimulation *in vitro*. RNAseq experiments showed that, except for C_μ_, C_H_ sense and antisense transcripts were dramatically reduced in 3′RR-deficient mice (Fig. 3a and Supplementary Figs 1–3). In contrast, E_μ_ deletion had no effect on C_H_ transcription at the IgH locus. RNASeq data are presented in a quantitative way with statistics in the Supplementary Fig. 4. Non coding RNAs (ncRNAs) play an important role in the targeting of the CSR machinery and contribute to chromosomal looping^10, 18^. Among these ncRNAs, enhancer RNA (eRNA) are transcribed from DNA sequences of enhancer including the 3′RR and contribute to their enhancer function^19, 20^, and chromosomal looping^4^. RNAseq experiments did not highlight any effect of E_μ_ deletion on both sense and antisense 3′RR eRNA levels (Fig. 2a). In contrast both sense and antisense transcription around the E_μ_ enhancer were consistently lowered in 3′RR-deficient activated B-cells. This decrease is reminiscent of the notable effect of the 3′RR SE deletion on μ transcription evidenced with quantitative PCR in resting mature B-cells^21^. Physical E_μ_-3′RR interactions were also documented in resting B-cells^4^. RNAseq data comparing splenic resting B-cells indicate that enhancer activity was, at this stage, already dominated by the 3′RR: while E_μ_ deletion showed no obvious effect on C_H_ transcription and 3′RR eRNAs, the 3′RR deletion does not modifies E_μ_ eRNA but still decreases μ transcripts and abrogates basal transcription of all downstream C_H_ (Fig. 2b). This confirmed a passive role for E_μ_ at the resting stage while H chain production originated from pV_H_ mostly rely on 3′RR, and more precisely on hs4^22^.

## Discussion

Studies highlighted different roles and kinetics of activation for IgH SEs during B-cell development. The E_μ_ SE regulates V(D)J recombination in pro-B cells^6, 7^, but is not crucial for SHM and CSR in mature B-cells^23^. The 3′RR SE regulates SHM^24^, and conventional CSR in mature B-cells^10, 25, 26^, but is dispensable for V(D)J recombination^27^. GFP transgenic mice reported that the E_μ_ SE is active at pro-B/pre-B cell stages^28^, while the 3′RR SE is active at immature/mature B-cell stages^29^. Despite different roles and kinetics, the IgH locus assumes in resting B-cells and CSR an enigmatic loop conformation with the E_μ_ enhancer and the 3′RR SE in close proximity^4^, suggesting a potential transcriptional cross-talk between these two enhancer entities. Present results strongly suggest that cross-talk is unidirectional. The 3′RR SE stands as a fully autonomous module in mature B-cells. The E_μ_ enhancer by contrast appears mostly dispensable at this stage, its deletion neither abrogating CSR-required sense/antisense germline transcription or 3′RR SE eRNA expression and activation-associated epigenetic mark enrichment. If the E_μ_ enhancer has no role on 3′RR activation during CSR, it itself shows at least partial 3′RR-dependence. Deletion of the 3′RR SE impacts E_μ_ H3K9ac activation-associated epigenetic marks and sense/anti-sense eRNA transcription.

During CSR the IgH locus assumes a loop conformation. While this conformation has obviously a major interest to simultaneously bring enhancer, SE, S regions and promoters into close proximity, our data suggest another unknown functional role, which is to place E_μ_ under the authority of the 3′RR SE. The E_μ_/S_μ_/S_x_/3′RR hub might have several successive purposes to facilitate CSR. This hub brings the S_x_ acceptor region in close proximity to the 3′RR SE, allowing for its efficient activation by acting notably on transcription and AID targeting^10^. It support physical/functional interactions between 3′RR elements hereby building their synergy^3^, and finally facilitates S_μ_-S_x_ synapsis, increasing the probability of a legitimate junction between donor and acceptor S regions and, in the meantime, reducing the risk of potentially oncogenic translocation^30–32^. Previously reported phenotypes of E_μ_- and 3′RR-deficient mice, now completed by the present study, show that both IgH enhancers can behave as completely autonomous elements with regards to chromatin marks, eRNA transcription and accessibility, with E_μ_ solely controlling early rearrangements of immature B-cells, and the 3′RR being both necessary and sufficient for late B-cell remodelling events. However in mature B-cells, this ends with E_μ_ falling under the control of the 3′RR SE and then undergoing 3′RR-dependent transcription and chromatin remodelling as almost all basic promoters of the locus. Finally, three SEs have been reported in pro-B cells: Eμ, 3′RR and an enigmatic region between C_γ1_ and C_γ2_^2, 11, 12^. Despite that 3′RR has little role on V(D)J recombination except for silencing early transcription in pro-B cells^33^, investigation of the cross-talk between these three SEs in pro-B cells would be of interest to reinforce our knowledge of their role at the immature B-cell maturation stage.

## Material and Methods

### Mice

129 *wt* mice (from Charles Rivers Laboratories, France), E_μ_MAR-deficient mice^7^, and 3′RR-deficient mice^25^ were used. E_μ_MAR-deficient mice and 3′RR-deficient mice were in a 129 background. Our research has been approved by our local ethics committee review board (Comité Régional d’Ethique sur l′Expérimentation Animale du Limousin, Limoges, France) and carried according the European guidelines for animal experimentation.

### Spleen cell cultures for B-cell activation

Single-cell suspensions of CD43^−^ spleen cells of *wt*, EμMAR-deficient mice and 3′RR-deficient mice (8–12 week old, male and female) were cultured at 1 × 10^6^ cells per ml in RPMI 1640 with 10% fetal calf serum with 5 μg per ml LPS with or without 20 ng/ml IL-4. Two days LPS-stimulated cells were used for RNAseq and S_μ_/S_γ3_ ChIP experiments. Two days LPS + IL4 stimulated cells were then used for S_μ_/S_γ1_ ChIP experiments. Four days LPS- and LPS + IL4 stimulated cells were used for γ3, γ2b and γ1 CSR flow cytometry analysis, respectively.

### Flow cytometry analysis

Cultured splenic B-cells were labelled with anti-B220-BV421, anti-IgG_1_-PE, anti-IgG_3_-FITC, anti-IgG_2b_-PE and anti-IgM-PC7 antibodies for 30 min at 4 °C. Labelled cells were analyzed on a Fortessa LSR2 (Beckman Coulter) with Kaluza.

### ChIP experiments

ChIP experiments were done on LPS- and LPS + IL4 stimulated CD43^−^ spleen cells as previously described^10^. In brief, 20 × 10^6^ B-cells were cross-linked at room temperature for 15 min in 15 ml PBS with 1% formaldehyde. The reaction was quenched with 0.125 M glycine. After lysis, chromatin was sonicated to 0.5–1 kb using a Vibracell 75043 (Thermo Fisher Scientific). After dilution in ChIP buffer (0.01% SDS, 1.1% Triton X-100, 1.2 mM EDTA, 16.7 mM Tris-HCl, pH 8.1, and 167 mM NaCl), chromatin was precleared by rotating for 2 h at 4 °C with 50 ml of 50% protein A/G slurry (0.2 mg per ml sheared salmon sperm DNA, 0.5 mg per ml BSA, and 50% protein A/G; Sigma). 1 × 10^6^ cell equivalents were saved as input, and 10 × 10^6^ cell equivalents were incubated overnight with anti-H3K4me3, anti-H3K9ac, anti-H3K27ac or control antibodies. Immune complexes were precipitated by the addition of protein A/G. Cross-linking was reversed by overnight incubation (70 °C) in TE buffer with 0.02% SDS and chromatin was phenol/chloroform extracted. Anti-H3K4me3 and antu-H3K9ac were obtained from Millipore (ref: 07473 and 06942) and anti-H3K27ac was obtained from Abcam (clone ab5131). PCR probes were the following: Eμ-forward: GGGAGTGAGGCTCTCTCATA; Eμ-reverse: ACCACAGCTACAAGTTTACCTA; hs3a-forward: GGGTAGGGCAGGGATGCTCACAT; hs3a-reverse: GCTCTGGTTTGGGGC ACCTGTGC; hs1,2-forward: AGCATAGGCCACTGGGACTGG; hs1,2-reverse: CTCTCA CTTCCCTGGGGTGTT; hs3b-forward: TGGTTTGGGGCACCTGTGCTGAG; hs3b-reverse: GGGTAGGGCAGGGATGTTCACAT; hs4-forward: CCATGGGACTGAAAC TCAGGGAACCAGAAC; hs4-reverse: CTCTGTGACTCGTCCTTAGG. PCR probes for S_μ_ C_μ_, S_γ1_, C_γ1_, S_γ3_ and C_γ3_ have been reported in a previous study^10^.

### RNAseq experiments

CD43^−^ splenocytes were obtained from 4 *wt*, 4 3′RR-deficient mice and 4 E_μ_MAR-deficient mice before and after 48 h of *in vitro* stimulation (1 × 10^6^ cells per ml in RPMI 1640 with 10% fetal calf serum) with 5 μg per ml LPS. RNA was extracted using miRNeasy kit from QIAGEN, according to the manufacturer instructions. Two pooled RNA (with two samples) were obtained for each genotype. RNA libraries were obtained using TruSeq Stranded Total RNA with Ribo-Zero Gold (Illumina), according to the manufacturer instruction. Libraries were sequenced on a NextSeq500 sequencer, using NextSeq 500/550 High Output Kit (Illumina). Illumina NextSeq500 paired-end 2 × 150 nt reads were mapped with STAR release v2.4.0a versus mm10 with gene model from Ensembl release 77 with default parameters. The long length of the reads allowed for their precise mapping on switch regions, previously reported as error prone with shorter reads due to the highly repetitive structure of these sequences^34^. Quantification of genes was then performed using feature Counts release subread-1.4.6-p1-Linux-x86_64 with “–primary -g gene_name -p -s 1 -M” options based on Ensembl GTF release 77 annotations.

### Accession number

Data were deposited in Gene Expression Omnibus under the accession number GSE90760.

## Electronic supplementary material

Supplemental material
