# Supplementary material for: Eμ and 3′RR IgH enhancers show hierarchic unilateral dependence in mature B-cells
Source: Sci Rep. 2017 Mar 27;7:442. doi: 10.1038/s41598-017-00575-0 (PMC5428668; doi:10.1038/s41598-017-00575-0)
Supplement: Supplementary file 1 — Supplemental material [file 41598_2017_575_MOESM1_ESM.pdf]

E_{μ} and 3'RR IgH enhancers show hierarchic unilateral dependence in mature B-cells

Saintamand A, Vincent-Fabert C, Marquet M, Ghazzaui N, Magnone V, Pinaud E, Cogné M, Denizot Y.

Supplemental legends to Figures

Supplemental Figure 1: RNAseq experiments with focus on I_{μ}, S_{μ}, C_{μ} and C_{δ} transcripts.

Supplemental Figure 2: RNAseq experiments with focus on $I_{\gamma 3}$, $S_{\gamma 3}$ and $C_{\gamma 3}$ transcripts.

Supplemental Figure 3: RNAseq experiments with focus on $I_{\gamma 2b}$, $S_{\gamma 2b}$ and $C_{\gamma 2b}$ transcripts.

Supplemental Figure 4: RNASeq experiments presented in a quantitative way with statistics. *p<0.05, **p<0.01, ***p<0.001 (FDR corrected *p* values).

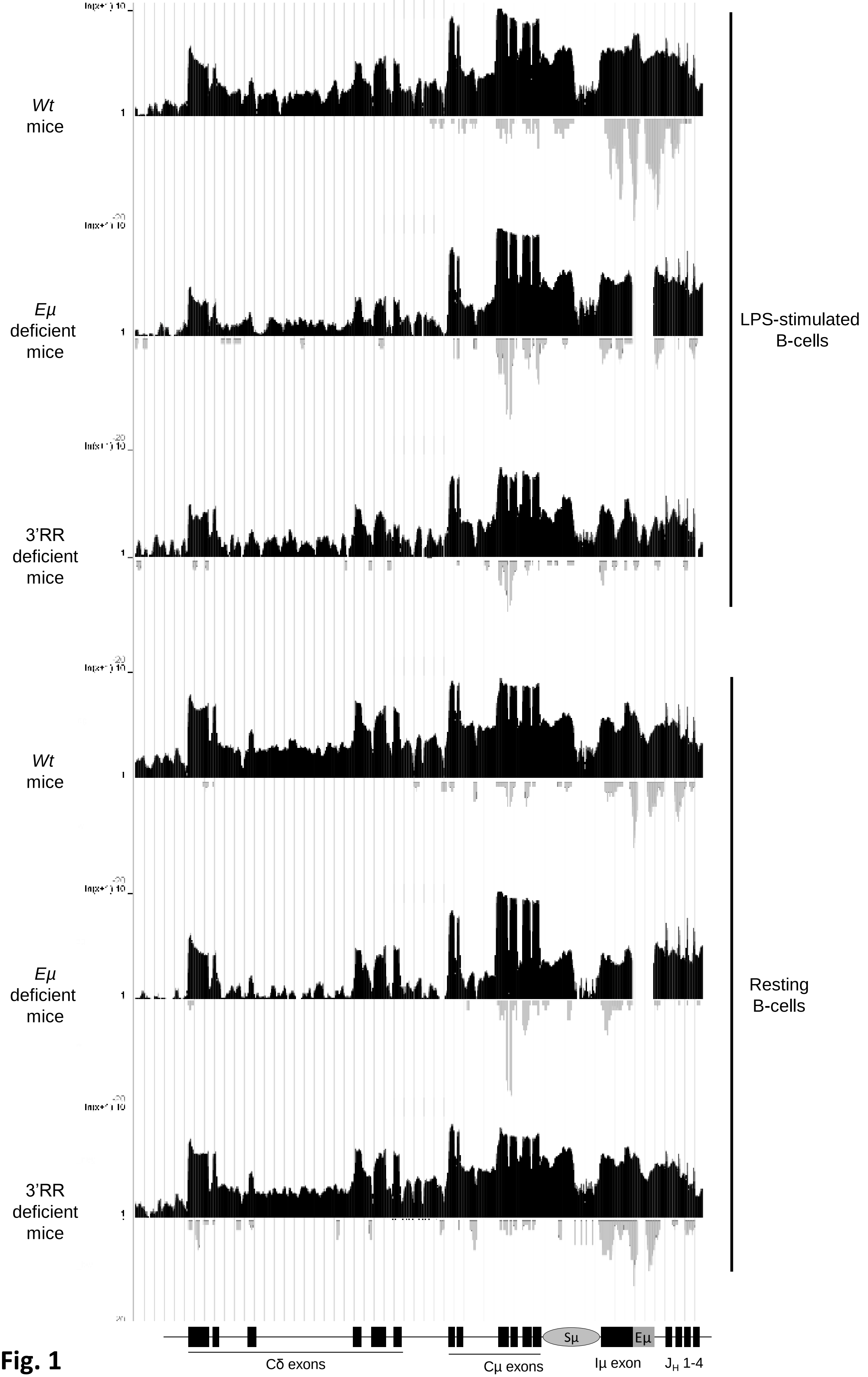

Wt mice
Eμ deficient mice
3'RR deficient mice
Wt mice
Eμ deficient mice
3'RR deficient mice
LPS-stimulated B-cells
Resting B-cells
Sμ
Eμ
Cδ exons
Cμ exons
Iμ exon
J_H 1-4

Supp. Fig. 1

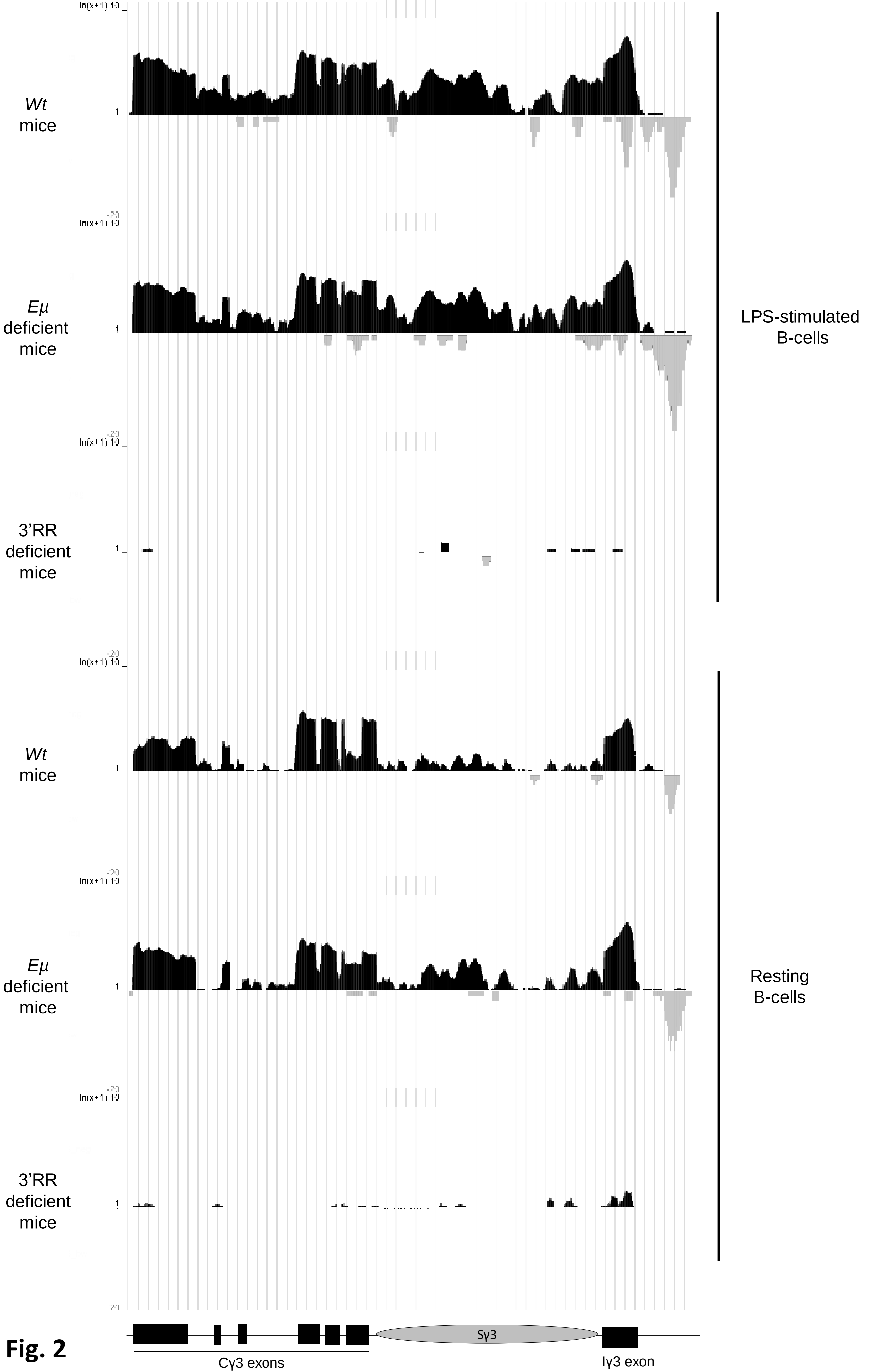
Wt mice
Eμ deficient mice
3'RR deficient mice
Wt mice
Eμ deficient mice
3'RR deficient mice
LPS-stimulated B-cells
Resting B-cells
Sγ3
Cγ3 exons
Iγ3 exon

Supp. Fig. 2

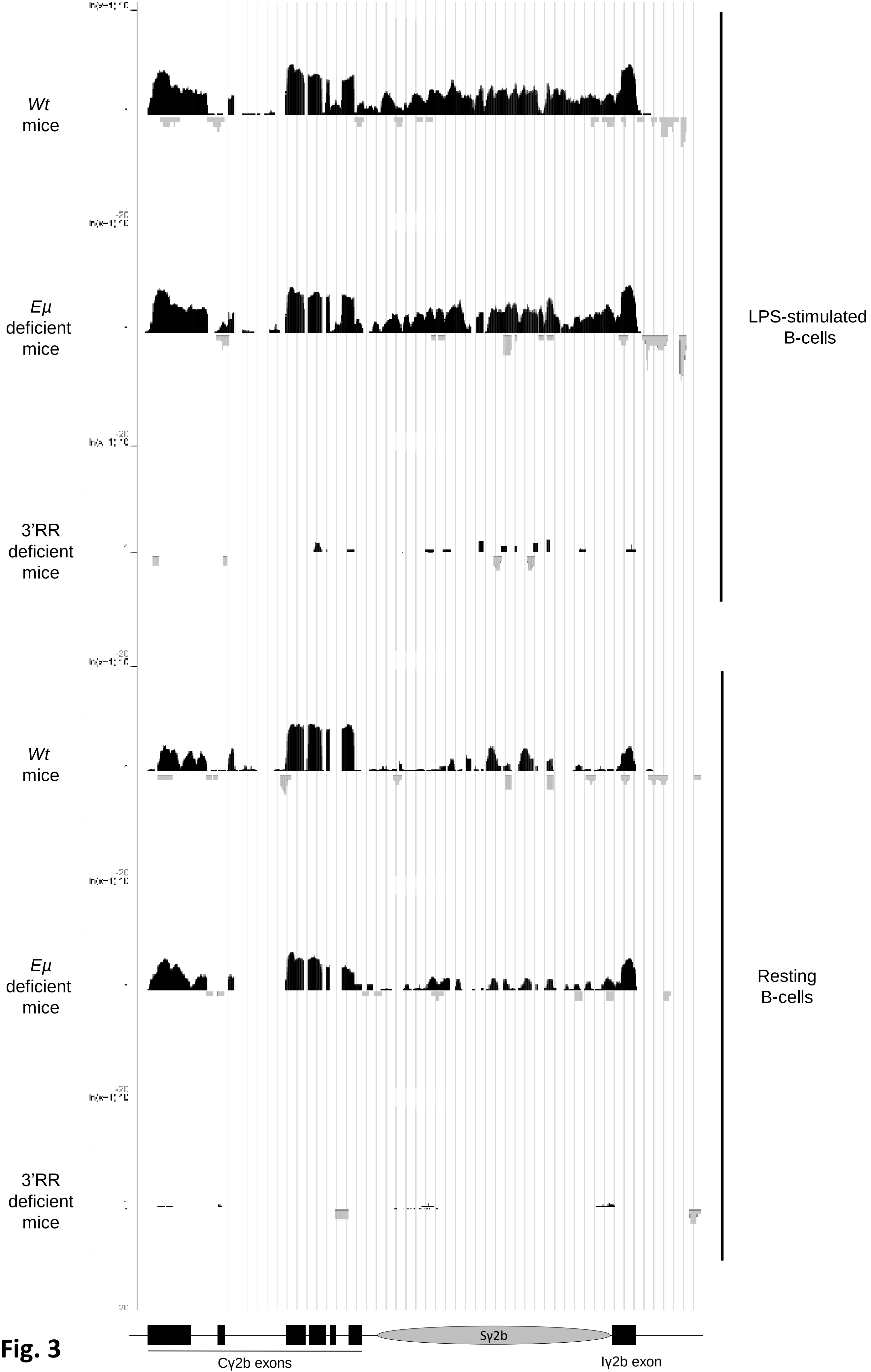
Wt
mice
Eμ
deficient
mice
3'RR
deficient
mice
LPS-stimulated
B-cells
Wt
mice
Eμ
deficient
mice
3'RR
deficient
mice
Resting
B-cells
Sγ2b
Cγ2b exons
Iγ2b exon

Supp. Fig. 3

A

LPS-stimulated B-cells

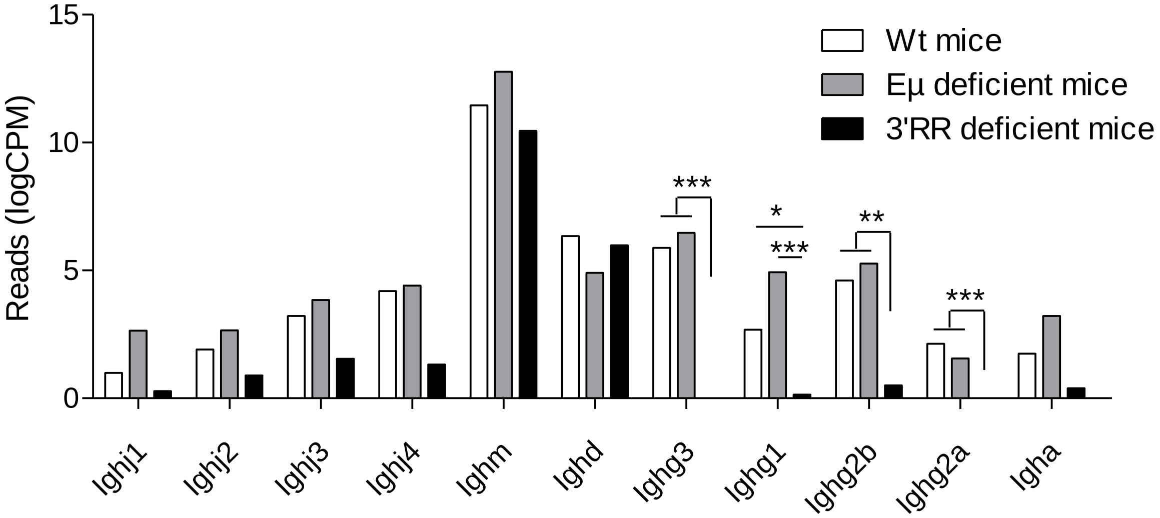

B

Resting B-cells

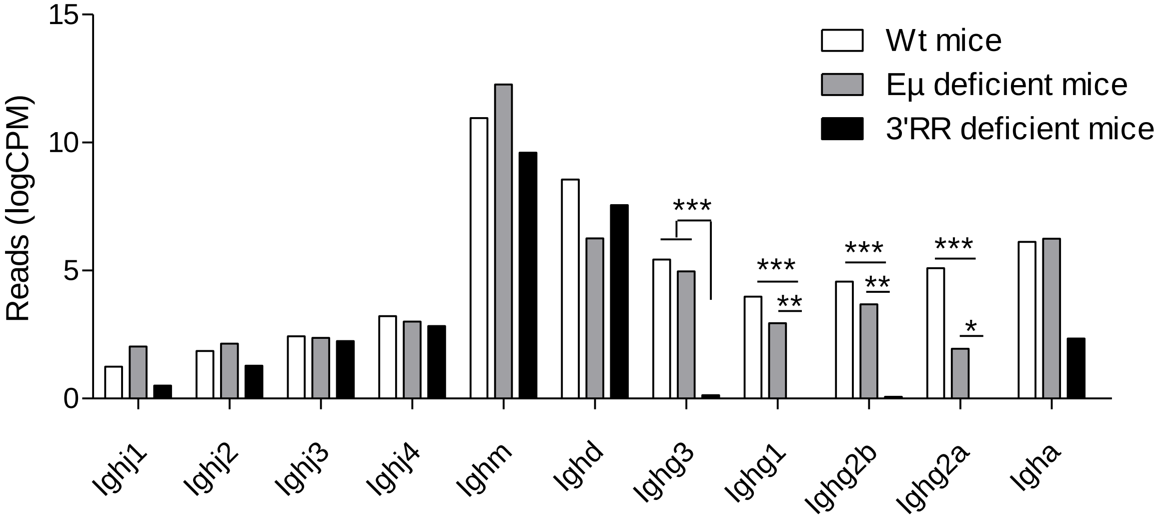

Supp. Fig. 4
